# Dietary Supplementation With Yucca Alleviates Heat Stress in Growing Broilers Exposed to High Ambient Temperature

**DOI:** 10.3389/fvets.2022.850715

**Published:** 2022-04-07

**Authors:** Jing Jing Luo, Wei Chen, Hao Qu, Yuan Qing Liu, Cheng Long Luo, Jian Ji, Ding Ming Shu, Jie Wang

**Affiliations:** ^1^Institute of Animal Science, Guangdong Academy of Agricultural Sciences, Guangzhou, China; ^2^State Key Laboratory of Livestock and Poultry Breeding, Guangzhou, China; ^3^Key Laboratory of Animal Nutrition and Feed Science in South China, Ministry of Agriculture, Guangzhou, China; ^4^Guangdong Public Laboratory of Animal Breeding and Nutrition, Guangzhou, China; ^5^Guangdong Key Laboratory of Animal Breeding and Nutrition, Guangzhou, China; ^6^Dekang Group Co., Ltd., Chengdu, China; ^7^Key Laboratory of Livestock and Poultry Breeding, Ministry of Agriculture, Guangzhou, China

**Keywords:** broiler, heat stress, yucca, feed intake, CCK, transient receptor potential vanilloid receptor

## Abstract

Yucca contains high a content of saponin that has a glucocorticord-like effect in animals, e.g., anti-inflammation and anti-microbiota. The objective of the present study was to test the hypothesis that dietary supplementation of yucca powder may alleviate heat stress and improve growth performance of growing broilers subjected to cycling high ambient temperature. A total of 240 male broiler chicks (yellow feathered chicken) aged 28 days, with body weight (BW) of 792 ± 43.7 g, were randomly allocated to one of four treatments (6 replicates per treatment): control (normal temperature, 24 ± 2°C, 24 h), fed diets supplemented with 100 mg/kg yucca under normal temperature (**Y**), high ambient temperature exposure (**HT**, 34 ± 2°C, 11 h), fed diets supplemented with 100 mg/kg yucca (**HT+Y**) under high ambient temperature. After 7 days of adaption, the experiment was conducted for 4 weeks (aged 28–56 days). HT significantly reduced feed intake, BW, and average daily gain (ADG) of broiler, but yucca improved the feed intake under HT condition. Yucca supplementation reduced (*P* < 0.05) the HT-induced increase in temperature of rectum and leg skin. Supplementation of yucca increased the hypothalamic mRNA expression of *TRPV2, TRPV4*, and *TRPM8* (*P* < 0.05). Yucca reduced (*P* < 0.05) the plasma lipid oxidation product malondialdehyde (**MDA**), but did not affect the activities of antioxidant enzyme superoxide oxidase (**SOD**) and glutathione peroxidase (**Gpx**). Yucca did not affect the plasma neuro peptide Y (**NPY**), which was reduced by HT, yucca reduced circulation cholecystokinin (**CCK**) and hypothalamic mRNA expression of *CCK*. Supplementation of yucca increased the mRNA expression of both heat and cool sensing receptors. The results of the present study indicate that yucca could improve antioxidant status and attenuate the heat stress response by regulating hypothalamic temperature-sensing genes in growing chickens. Besides, yucca supplementation improved feed intake probably through modulating CCK in growing broilers under high ambient temperature.

## Introduction

For poultry production during summer, the compromised performance of birds causes great economic loss, which increasingly becomes a great concern worldwide as the global climate changes. Poultry are air temperature-sensitive and the elevation of ambient temperature can disrupt the normal physiological behavior and cause heat stress, most obviously reflected in the increased breathing rate and reduced feed intake (1, 2). Under heat stress, in order to adapt the high ambient temperature (HT), birds reduce feed consumption to reduce heat production. To date, the exact mechanism on the HT induced-reduction of feed intake remains unclear, but it has been assumed to be related to the hypothalamic-pituitary-thyroid axis (3). The mechanism of food intake regulation has been well-demonstrated in mammals as food intake is a complex biological process, involving in both the peripheral and central nervous system **(CNS)** control mechanisms (4). Like in mammals, the CNS, specifically the hypothalamus, has emerged as a key site in appetite control for birds (5), and are potentially involved in the regulation of birds' feed intake under stress.

Researchers have been exploring available ways to alleviate the negative effects of heat stress on poultry production. Dietary supplementation of additives with biological activities are of great concern among these alternatives because of its practical application. Yucca schidigera (YS, Agavaceae) is a species of plant prevalent in the Americas, especially the southwestern United States and northern Mexico (6). Yucca schidigera contains a variety of phytochemicals such as steroid saponins, glycol, poly phenols, and resveratrol (7). Since saponin molecules own a similar molecular structure with glucocorticord, the saponin exerts a glucocorticord-like effect through glucocorticord receptor (**GR**), e.g., antimicrobial (8, 9), antioxidant (10, 11), anti-inflammatory (12), and immunomodulatory properties (13, 14). Also, saponin has been demonstrated to be an effective anti-depressant in animals (15). However, whether yucca could attenuate heat stress and improve feed intake of broilers under high ambient temperature remains unknown. The objective of the present study was, therefore, to test the hypothesis that dietary supplementation of yucca may attenuate the heat stress in broilers and improve feed intake under high ambient temperature, and potential mechanism were thereafter explored by evaluating the feed intake-related hormones in hypothalamus, circulation, and intestine. These expected results have significant implications for modulating heat stress.

## Materials and Methods

### Ethics Statement

All animal experimental procedures were performed according to Regulations for the Administration of Affairs Concerning Experimental Animals of the State Council of the People's Republic of China and authorized by Animal Welfare Committee of Institutes of Animal Sciences, Guangdong Academy of Agricultural Sciences (IASCAAS2022).

### Animals and Experimental Design

A total of 240 male broiler chickens (yellow feathered chicken, typical broiler breeder in South China) aged 21 days, with average BW of 542 ± 61 g, were purchased from Guangdong Zhiwei Agricultural Technology Co., Ltd. (Guangdong, China). Birds were caged (two birds per cage) in an environmentally-controlled room for 7 days of adaption (relative humidity, 60%; temperature, 24 ± 2°C). Individual cages were 30 cm wide × 35 cm deep × 35 cm high. At day 28, birds were weighed and marked individually and then randomly allocated to 4 treatments with 6 replicates of 10 chickens, were caged (two birds per cage), and were subjected to the following treatments: (1) control (**CON**), room temperature was kept at 24°C and 60% humidity for 24 h; (2) fed diets supplemented with 100 mg/kg yucca powder under room temperature (**Y**); (3) exposed to high ambient temperature (HT) for 11 h (34 ± 2°C, 80% humidity, 8:00 am−19:00 pm per day), and during the other 13 h they were kept at room temperature (24 ± 2°C, 60% humidity, 19:00 pm−8:00 am the next day); (4) fed diets supplemented with 100 mg/kg yucca powder under high ambient temperature (**HT+Y**). Experiment was conducted for 4 weeks (aged 29–56 days). The yucca powder product (DK Sarsaponin 40) was provided by Desert King Company (Baja California, Mexico), and the dose of yucca supplementation (100 mg/kg) was recommended by this company based on previous studies on yucca (16, 17). According to AOAC method of proximate analysis (18), the yucca powder contains 4.5% moisture, 3.2% crude protein, 26.7% crude fiber and 9% ash. After extraction of saponins with the method of Soxhlet reflux extraction (19), the analyzed saponins content of yucca powder was 12.5% for UV spectrometer and 3% for HPLC method, respectively. Experimental diets (Table 1) were formulated to meet the nutrient requirement for broilers recommended by National Research Council (20). Broilers had free access to feed and water and subjected to 16 h of light per day.

**Table 1:** Composition and nutrient levels of the basal diet (air-dry basis, %).

**Ingredients**	**%**
Corn	70.40
Soybean meal	16.75
Wheat bran	6.00
Soybean oil	3.00
Lys.HCl	0.19
Met	0.13
Thr	0.10
CaHPO_4_	1.60
Limestone	0.53
NaCl	0.30
Premix^a^	1.00
Total	100.00
**Calculated nutrient levels**	
Metabolizable energy, MJ/kg	12.89
Crude protein, %	17.56
Ether extract, %	2.54
Ash, %	4.75
Calcium, %	0.87
Total phosphorus, %	0.40
Lys, %	0.81
Met, %	0.35
Met+Cys, %	0.58
Trp, %	0.15
Thr, %	0.63
Arg, %	0.91

a *Premix provided per kilogram of diet: D-pantothenic acid, 10.9 mg; folic acid, 0.95 mg; nicotinic acid, 30 mg; biotin, 0.16 mg; vitamin A, 8,000 IU; vitamin B_1_, 1.7 mg; vitamin B_2_, 8.2 mg; vitamin B_6_, 2.78 mg; vitamin B_12_, 0.015 mg; vitamin D, 2,800 IU; vitamin E, 30 IU; vitamin K_3_, 3.32 mg; Zn, 68 mg; Fe, 81 mg; Mn, 82 mg; I, 0.5 mg; Cu, 9 mg; Se, 0.27 mg*.

### Performance, Body Temperature Measurement, and Sample Collection

*Ad libitum* feed was provided daily and feed refusals were recorded weekly, which were used in calculating the average daily feed intake on per replicate basis. Boiler chickens from all treatments were weighed at the 1st week (aged 35 days), 2nd week (aged 42 days), 3rd week (aged 49 days), and 4th week (aged 56 days) of the experimental period after 12 h of fasting. The average daily gain (**ADG**) and feed conversion ratio (**FCR**) of each week were calculated accordingly.

Rectal temperatures were recorded according to the method of Farghly et al. (21), with minor modifications. Briefly, two chickens were randomly selected from each replicate at day 53. A digital thermometer (Braun, model Prt1000) was inserted into the cloaca for 2 min at 3 cm depth in the afternoon (between 14:00 and 15:00), rectal temperatures were then recorded individually. Skin temperature at both of the left and right leg was measured using an infrared temperature gun (Raytek, model MiniTemp MT6).

At the end of experiment (day 56), one bird from each replicate was randomly weighed and selected for sample collection after 12 h of fasting. At 8:00 am of each sampling day, five milliliters of blood were drawn from the wing vein and collected in non-heparinized collecting tubes. After centrifugation at 3, 000 × g, 4°C for 10 min, serum was collected with six aliquots and stored at −80°C. After blood sampling, broilers were killed by cervical dislocation. Hypothalamus and duodenal tissues were harvested, immediately frozen in liquid nitrogen, and stored at −80°C until subsequent analysis.

### Serum Biochemistry

Serum concentration of corticosterone, ghrelin, NPY, and CCK were determined using ELISA kit, according to the manufacturer's instructions (MEIMIAN, Jiangsu Feiya Biological Technology Co. Ltd., Jiangsu, China). Serum superoxide oxidase (**SOD**), glutathione peroxidase (**Gpx**) and malondialdehyde (**MDA**) were analyzed using commercial kit, according to the manufacturer's instructions (JIANCHENG, Nanjing Jiancheng Biotechnology Co. Ltd., Nanjing, China).

### Tissue RNA Extraction and Real-Time Quantitative PCR

Total RNA was extracted from hypothalamus and duodenum tissues using Trizol reagent according to the manufacturer's instructions (Invitrogen, Carlsbad, CA). RNA quality was determined by examining the absorption rate at 260 nm and 280 nm with UV spectrophotometry. After RNA was treated with DNAase, cDNA was then transcribed via reverse transcription kits (PrimeScript RT reagent Kit with gDNA Eraser, RR047 A, Takara, Dalian, China).

The mRNA abundance of the target gene in hypothalamic and duodenum were detected by the real-time quantitative PCR. Real-time PCR reaction system was composed of a total volume of 20 μL containing 1 μL of the cDNA product, 10 μL of SYBR-green PCR master Mix (TB Green Premix ExTaq, RR420 A, TAKARA), and 0.2 μmol/L of gene-specific forward and reverse primers (Table 2). Real-time PCR reactions were carried out on the CFX96 Touch Real Time PCR Detection System (BIO-RAD, Hercules, CA) according to the following steps: 5 min at 95°C, 39 cycles of denaturation at 95°C for 30 s, annealing at 58°C for 30 s, and extension at 72°C for 30 s. The relative fold change was performed using the 2^−ΔΔCt^ method with *GAPDH* as an internal control.

**Table 2:** Specific gene primers used for real-time quantitative PCR.

**Gene**	**Accession number**	**Primers sequences**	**Product length, bp**
*GAPDH*	NM_204305.1	F:GGTGAAAGTCGGAGTCAACGG	108
		R:TCGATGAAGGGATCATTGATGGC	
*CCK*	NM_001001741	F:CAGCAGAGCCTGACAGAACC	121
		R:AGAGAACCTCCCAGTGGAACC	
*POMC*	NM_001031098	F:ATCAAGGTGTACCCCAACGG	300
		R:CCTTCTTGTAGGCGCTTTTG	
*Ghrelin*	AB075215	F:CCTTGGGACAGAAACTGCTC	203
		R:CACCAATTTCAAAAGGAACG	
*TRPV1*	395259	F:TGGGACCGATTTGTCAAGCA	126
		R:AAAAGCGAAGGGAGGCTTGT	
*TRPV2*	XM_004946685.4	F:CTGAACTATCGGCCTGGACT	224
		R:TCTTCCCCGTCTTTGCATCT	
*TRPV3*	771945	F:TTACTGTTACTGAGGCTGAAGGC	314
		R:GCCTTACAGGGTTTCTTACATCTTT	
*TRPV4*	NM_204692.1	F:CCTGGTGATGATTGCAGATG	176
		R: GGTGCCTGTACACTGGGTCT	
*TRPM8*	XM_027461829.2	F:AGTGGAACCAACTGGACCTG	234
		R:TTGCAATCTGCAGGTTCTTG	

*GAPDH, glyceraldehyde-3-phosphate dehydrogenase; CCK, cholecystokinin; POMC, proopiomelanocortin; TRPV1–4, transient receptor potential vanilloid receptor type 1–4; TRPM8, transient receptor potential melastatin type 8*.

### Statistical Analysis

Data for a 2 × 2 factorial arrangement of treatments, with the main effects for ambient temperature (24°C vs. 34°C) and yucca (100 mg/kg yucca vs. no yucca) and the interaction effects between temperature and yucca were analyzed using general linear model procedures for analysis of variance (ANOVA) (22). A replicate was the experimental unit for the growth performance data, while each replicate was the experimental unit for other measured parameters (plasma biochemical variables, mRNA expression). The statistical model included treatment, replicate, and all two- and three-way interactions as sources of variation. Broilers with treatment × replicate were used as the random variable in the model. The parameters for growth performance (BW, ADG, ADFI, and FCR), temperature of rectum and leg skin, plasma biochemistry (MDA, GSH, antioxidant enzyme, hormone), and gene mRNA expression were analyzed using two-way ANOVA with repeated measures. The results are expressed as the mean ± SEM of measurements on tissues from 6 replicate broilers at each group setting *P* < 0.05 as a criterion of statistical significance.

## Results

**Figure 1:**
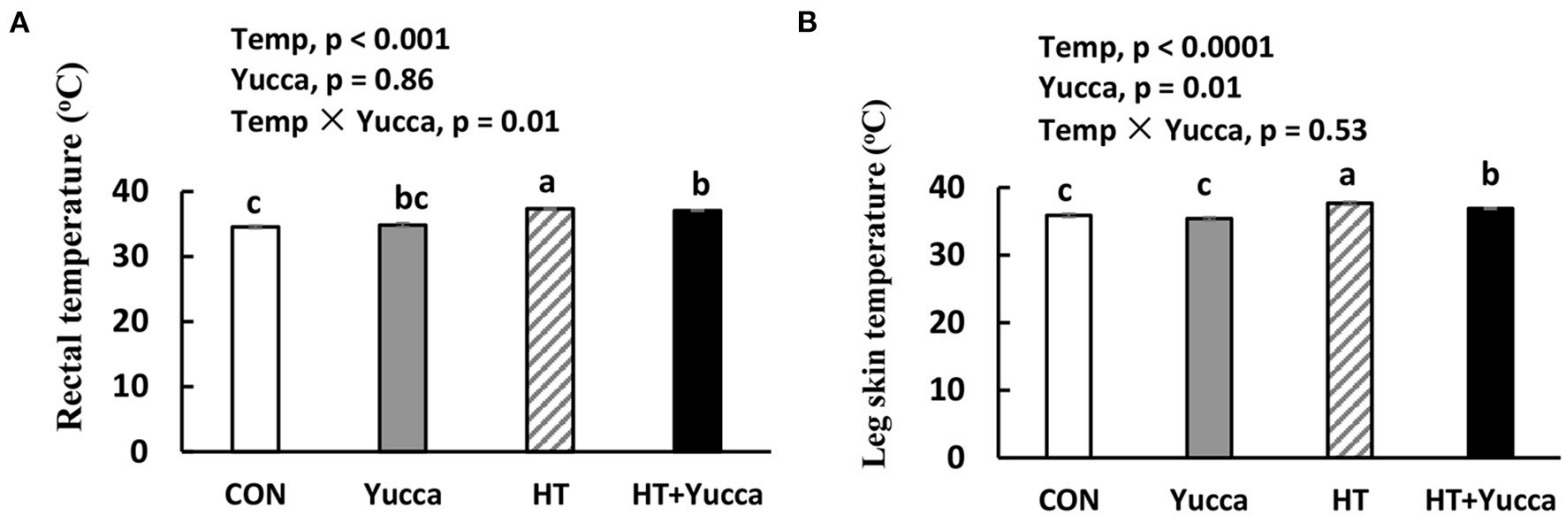
Rectal temperature **(A)** and leg skin temperature **(B)** in broilers at 56 days. Broilers, of 28 days of age, were fed a basal diet and exposed to 24°C (CON) or 34°C (HT), or a basal diet supplemented with 0.1% yucca and exposed to 24°C (Yucca) or 34°C (HT + Yucca) for 4 wks. Values are means ± SE, *n* = 6. Means without a common letter differ (*P* < 0.05).

**Figure 2:**
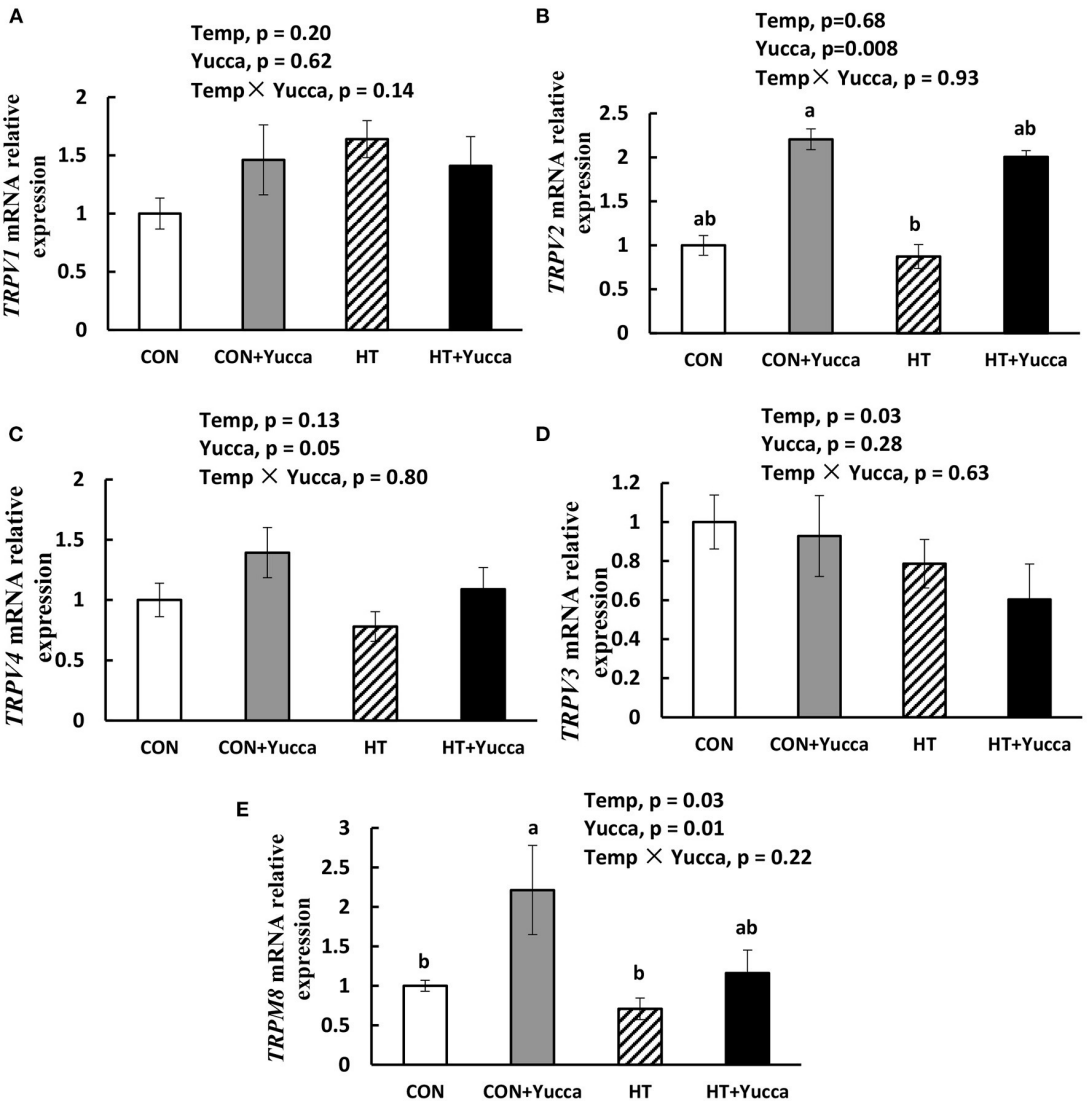
Hypothalamic mRNA expression of *TRPV1* **(A)**, *TRPV2* **(B)**, *TRPV4* **(C)**, *TRPV3* **(D)**, and *TRPM8* **(E)** in broilers at 56 days. Broilers, of 28 days of age, were fed a basal diet and exposed to 24°C (CON) or 34°C (HT), or a basal diet supplemented with 0.1% yucca and exposed to 24°C (Yucca) or 34°C (HT + Yucca) for 4 wks. Values are means ± SE, *n* = 6. Means without a common letter differ (*P* < 0.05).

As shown in Table 3, **HT** significantly reduced feed intake, BW, and ADG of broilers during each growth period except for the first week of the experiment (*P* < 0.05). Also, HT increased FCR (*P* < 0.05) during each week of the experiment. There were interaction effects of ambient temperature and yucca supplementation on the broiler's feed intake during the entire experimental period (28 days−56 days). The temperature of both rectum and leg skin in broilers increased by HT (increasing approximately by 0.7 or 1.8°C, for rectum and leg skin, respectively, Figures 1A,B), but they were reduced by yucca supplementation (*P* < 0.01). There were interaction effects of ambient temperature and yucca on the rectal temperature of broilers at 56 days. The hypothalamic mRNA expression of *transient receptor potential vanilloid receptor 1* (***TRPV1***) was not affected by HT or yucca (Figure 2A), while supplementation of yucca increased the hypothalamic mRNA expression of *TRPV2* (Figure 2B) and *TRPV4* (Figure 2C). The HT decreased the mRNA expression of both *TRPV3* (Figure 2D) and *transient receptor potential melastatin type 8* (***TRPM8)***, but yucca supplementation increased the mRNA expression of *TRPM8* (*P* < 0.05, Figure 2E).

**Table 3:** Effects of high ambient temperature and yucca on the growth performance of broiler chickens.

	**24** **°** **C**		**34** **°** **C**		**SEM**	***P*** **-value**		
	**–Yucca**	**+Yucca**	**–Yucca**	**+Yucca**		**T**	**Y**	**T × Y**
**BW**								
Initial (28 d)	785	789	791	806	8.46	–	–	–
29–35 d	1,015	1,015	1,000	1,019	11.4	ns	ns	ns
36–42 d	1,331	1,323	1,268	1,269	16.2	0.0005	ns	ns
43–49 d	1,676	1,655	1,533	1,540	26.3	<0.0001	ns	ns
50–56 d	1,982	1,886	1,794	1,853	406	0.01	ns	0.07
**ADG, g/d**								
29–35 d	32.7	32.2	29.8	30.4	0.868	0.007	ns	ns
36–42 d	44.4	44.5	38.0	36.8	0.910	<0.0001	ns	ns
43–49 d	48.8	49.3	38.9	39.6	1.25	<0.0001	ns	ns
50–56 d	45.6	42.8	35.7	36.2	1.77	<0.0001	ns	ns
29–56 d	42.1	40.3	35.4	37.9	1.28	0.001	ns	ns
**ADFI, g/d**								
29–35 d	80.5	80.1	73.3	74.7	1.22	<0.0001	ns	ns
36–42 d	106	103	97.5	98.1	2.17	0.004	ns	ns
43–49 d	123	123	107	102	3.00	<0.0001	ns	ns
50–56 d	121	116	107	115	3.47	0.04	ns	0.09
29–56 d	107	102	97.8	102	2.23	0.04	ns	0.04
**FCR**								
29–35 d	2.48	2.54	2.48	2.53	0.069	ns	ns	ns
36–42 d	2.46	2.35	2.57	2.65	0.047	<0.0001	ns	0.05
43–49 d	2.52	2.46	2.73	2.60	0.048	0.0005	0.04	ns
50–56 d	2.67	2.67	2.93	3.08	0.096	0.003	ns	ns
29–56 d	2.56	2.55	2.78	2.72	0.070	0.01	ns	ns

*T, ambient temperature; Y, yucca; ns, no significant; n = 6*.

*–Yucca, basal diets without yucca; +Yucca, basal diets with supplementation of yucca*.

**Figure 3:**
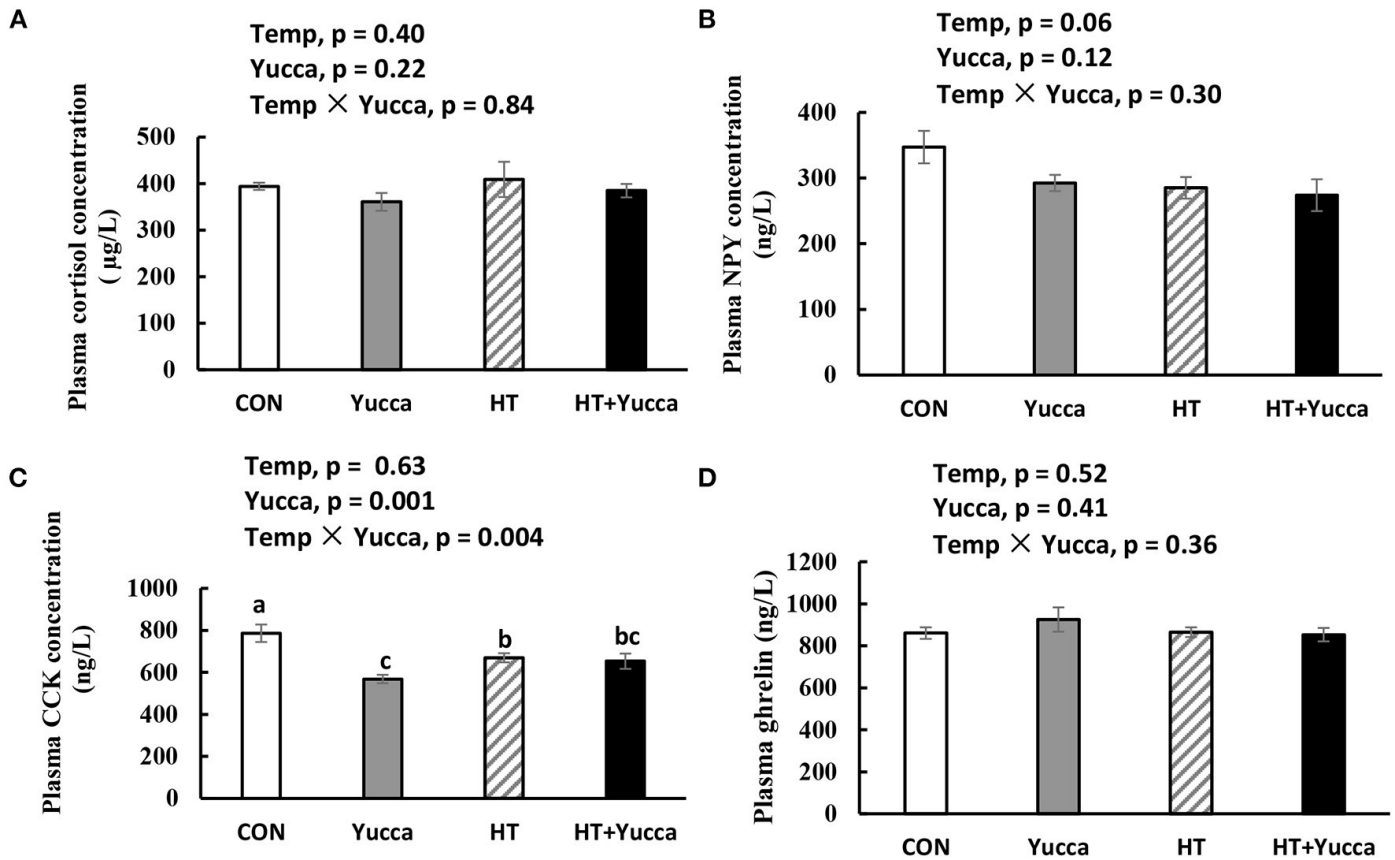
Plasma concentration of cortisol **(A)**, NPY **(B)**, CCK **(C)**, and ghrelin **(D)** in broilers at 56 days. Broilers, of 28 days of age, were fed a basal diet and exposed to 24°C (CON) or 34°C (HT), or a basal diet supplemented with 0.1% yucca and exposed to 24°C (Yucca) or 34°C (HT + Yucca) for 4 wks. Values are means ± SE, *n* = 6. Means without a common letter differ (*P* < 0.05). CCK, cholecystokinin; NPY, neuropeptide.

The plasma concentration of cortisol in broilers was not affected by HT for 28 days (Figure 3A). Plasma NPY concentration was reduced by HT (*P* = 0.06, Figure 3B), but it was not affected by yucca supplementation. The plasma concentration of CCK was not affected by HT, but yucca supplementation reduced it (Figure 3C). Neither HT nor yucca did not affect plasma ghrelin (Figure 3D).

**Figure 4:**
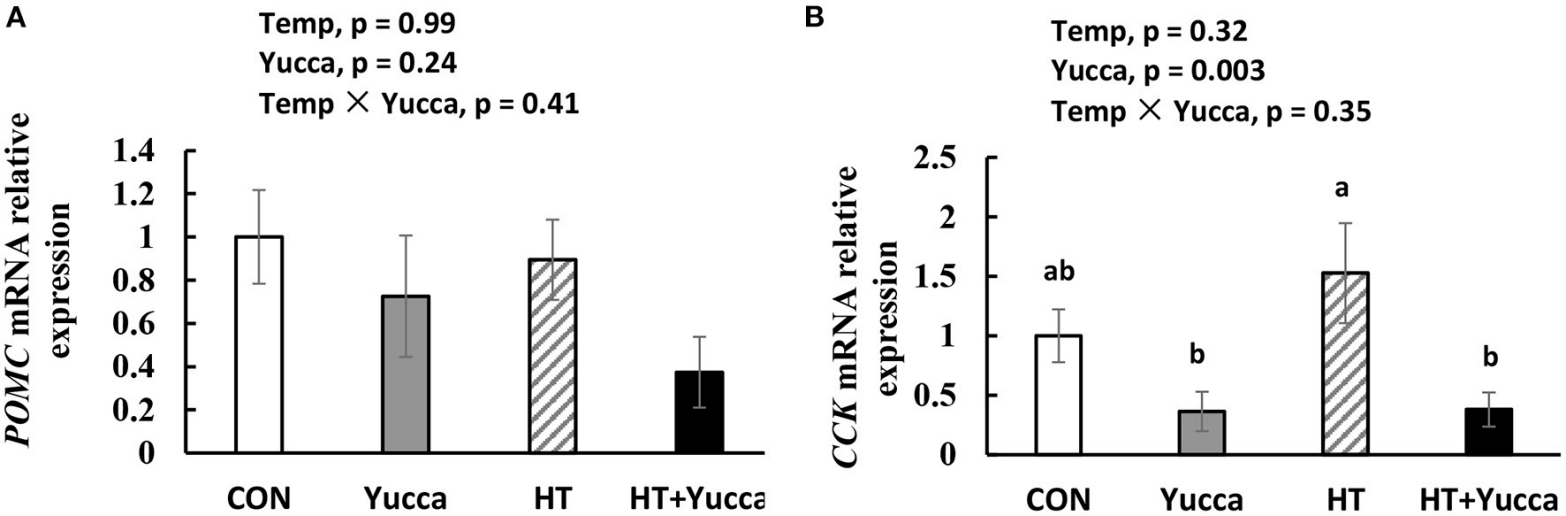
Hypothalamic mRNA expression of *POMC* **(A)** and *CCK* **(B)** in broilers at 56 days. Broilers, of 28 days of age, were fed a basal diet and exposed to 24°C (CON) or 34°C (HT), or a basal diet supplemented with 0.1% yucca and exposed to 24°C (Yucca) or 34°C (HT + Yucca) for 4 wks. Values are means ± SE, *n* = 6. Means without a common letter differ (*P* < 0.05).

**Figure 5:**
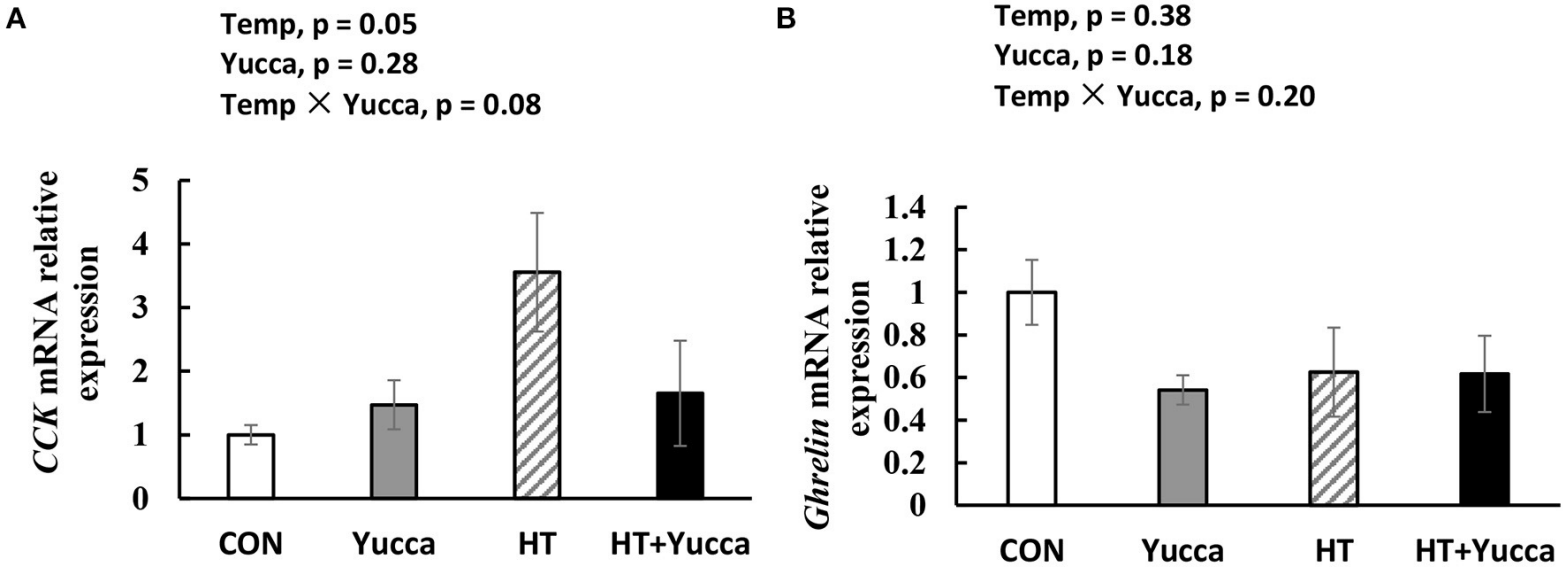
Duodenal mRNA expression of *CCK* **(A)** and *Ghrelin* **(B)** in broilers. Broilers, of 28 days ofage, were fed a basal diet and exposed to 24°C (CON) or 34°C (HT), or a basal diet supplemented with 0.1% yucca and exposed to 24°C (Yucca) or 34°C (HT + Yucca) for 4 wks. Values are means ± SE, *n* = 6.

Hypothalamic mRNA expression of *CCK* in broilers were significantly increased (*P* < 0.05) by HT, while supplementation with yucca reduced it (Figure 4A). Hypothalamic mRNA expression of *POMC* was not affected either by HT or yucca supplementation (*P* > 0.05, Figure 4B). The duodenal mRNA expression of *CCK* was increased by HT (*P* = 0.05, Figure 5A), and yucca supplementation had no effect on the duodenal mRNA expression of *CCK*. Neither HT nor yucca supplementation exerted effects on duodenal mRNA expression of *ghrelin* (*P* > 0.05, Figure 5B).

**Figure 6:**
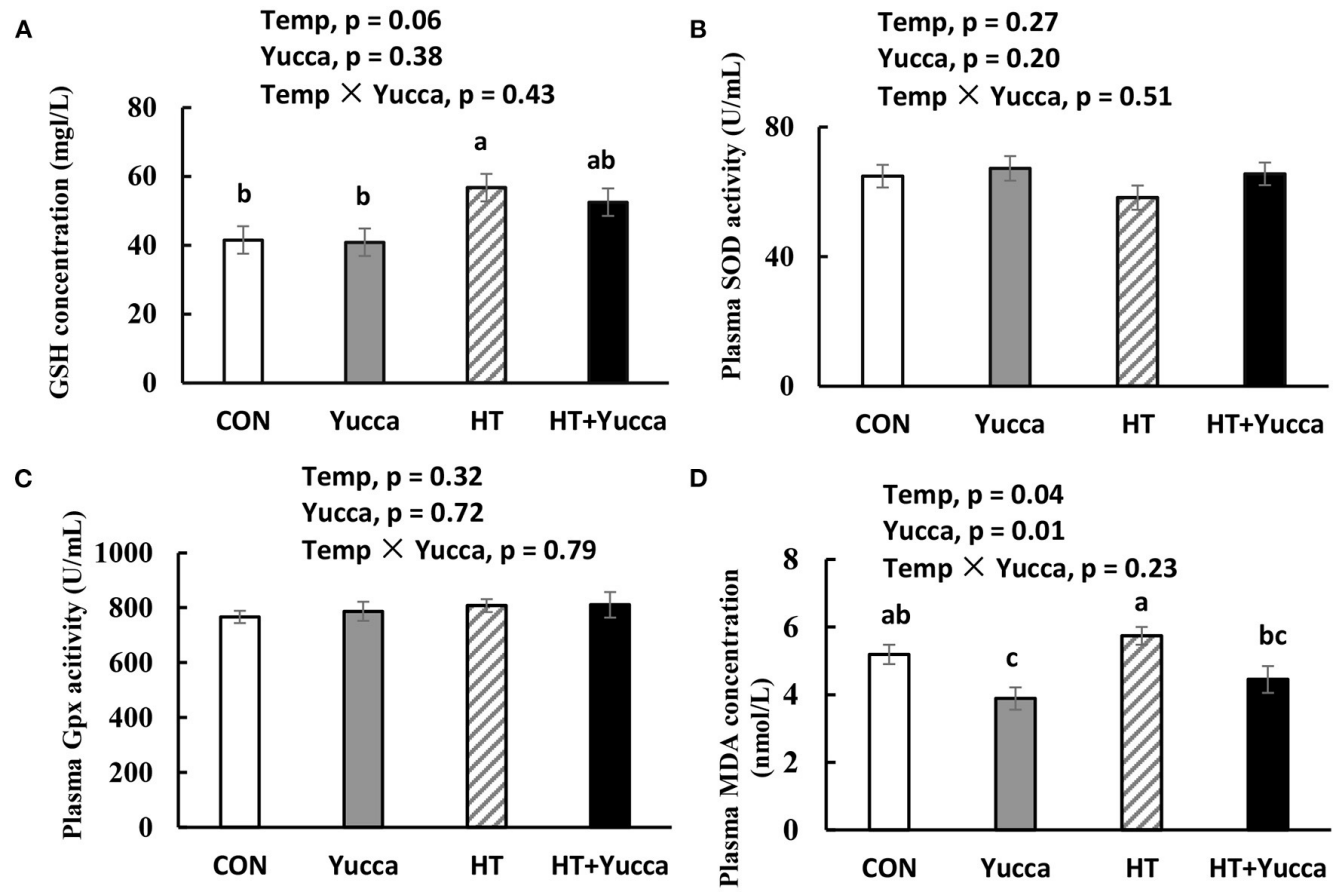
Concentration of GSH **(A)**, activities of SOD **(B)** and Gpx **(C)** and concentration of MDA **(D)** in serum of broilers at 56 days. Broilers, at 28 days of age, were fed a basal diet and exposed to 24°C (CON) or 34°C (HT), or a basal diet supplemented with 0.1% yucca and exposed to 24°C (Yucca) or 34°C (HT + Yucca) for 4 wks. Values are means ± SE, *n* = 6. Means without a common letter differ, *P* < 0.05. Gpx, glutathione peroxidase; MDA, malondialdehyde; SOD, superoxide dismutase.

The plasma concentration of GSH was increased by HT (*P* < 0.05, Figure 6A), whereas, the activities of antioxidant enzymes SOD and Gpx were not affected by HT (*P* > 0.05, Figures 6B,C). The plasma concentration of MDA in broilers was increased by HT (*P* < 0.05, Figure 6D), while yucca supplementation reduced it (*P* < 0.05, Figure 6D).

## Discussion

In this study, raising the temperature from 24 to 34°C caused heat stress to broilers, as reflected by the increased body temperature and breathing rate (observation). Specially, the feed intake of broilers was obviously reduced by HT, accompanied with the reduced daily gain, as well as decreased FCR during each week of the experiment. These observations are in accordance with previous studies in broilers (23, 24) and ducks (2). In this study, the dietary supplementation of yucca under heat stress attenuated the reduced feed intake of broiler chickens especially during the late stage of HT treatment, which thereafter resulted in a slight increase in ADG. Similarly, previous studies have also shown that yucca supplementation could improve feed conversion and growth of chicks (25). These positive effects are probably due to the relatively high content of saponin (12.5% for UV method) in yucca powder. Saponin has been demonstrated to be an effective anti-depressant in animals (15). For example, pretreatment of ginseng saponin to mice by intraperitoneal injection (5 or 20 mg/kg BW) attenuated the adrenocorticotrophic hormone (ACTH)-induced stress (26).

Interestingly, yucca supplementation prevented the HT-induced increase of rectal and leg skin temperature, indicating the alleviation effects of heat stress by yucca. The homeostatic control of body temperature is essential for the survival of mammal and birds and is known to be regulated in part by temperature sensitive neurons in the hypothalamus. Previous studies have shown that the preoptic area (POA) of the hypothalamus plays an important role in maintaining a stable Tcore via afferent inputs from skin thermoreceptors. The direct sensing of changes in skin temperature, in turn, activates POA efferent signals that control thermal effector organs (27, 28). The transient receptor potential (TRP) family ion channels have shown to be involved in thermosensation and are located in sensory nerves and skin. TRPV1, TRPV2, TRPV3, and TRPV4 can respond to heat at different temperature thresholds, while TRPM8 is responsive to cool or cold temperatures (15–30°C) [for more detail see review of Wetsel, (29)]. Interestingly, in this study, the high temperature decreased the hypothalamic mRNA expression of *TRPM8*, while supplementation of yucca increased the mRNA expression of both heat (*TRPV2, TRPV4*) and cool (*TRPM8*) sensing receptors. This indicates the increased thermoregulation capacity of yucca under heat stress, and thereafter attenuated the increased body temperature induced by HT. However, it remains unclear how yucca regulates the hypothalamic genes of heat and cool sensing receptor, which needs to be explored in future research.

Moreover, we found here that, under HT conditions, yucca powder improved the feed intake of broilers while no effects were observed under normal temperature, suggesting the interactive effects of yucca and ambient temperature in affecting feed intake. Specifically, yucca supplementation attenuated the reduction of feed intake induced by HT during the late period of the experiment, suggesting the time-dependent role of yucca in modulating feed intake of broilers under heat stress. It was reported that under normal temperature conditions, dietary supplementation of yucca schidigera powder have no effect on feed intake in adult laying hens (30), and some even reported to have negative effects on feed intake in growing broilers (17) because of the astringent and irritating taste of saponin. It is of great interest to explore the mechanism by which yucca exerted positive effects on the feed intake of broiler chickens under stress. In birds, the hypothalamus plays a vital role in integrating external environmental cues and generates the appropriate responses to influence feed intake (31). Hypothalamic neurons can perceive the increase in body temperature and exert an inhibiting influence on cells that are responsible for controlling feed intake (32), mainly through producing both orexigenic (agouti-related protein and neuropeptide Y, NPY) and anorexigenic (e.g., POMC) peptides (33). Besides, a number of circulating factors produced by peripheral organs are important in regulating feed intake, for example, leptin by adipose tissue, insulin and pancreatic polypeptide by the pancreas, gut hormones (e.g., ghrelin, peptide YY), and triiodothyronine by the thyroid gland. In this study, raising the temperature from 24 to 34°C significantly reduced the feed intake of broilers. This is probably due to the HT induced reduction in plasma orexigenic peptide (NPY), as well as up-regulation of *CCK* mRNA expression both in the hypothalamus and intestine. Similarly, it was reported that the concentrations of cholecystokinin (CCK) were reported to be increased both in plasma and intestine on 35 or 42-day-old broilers under HT condition (24). CCK is an anorexigenic peptide in poultry (31), able to slow the passage rate of feed in the gut (34), therefore exerting negative effects on feed intake. Our results demonstrated that the changes in plasma or hypothalamic CCK and NPY probably plays a role in suppressing feed intake under HT conditions. The improvement in feed intake by yucca was probably due to down-regulated mRNA expression of hypothalamic anorexigenic factors genes CCK and POMC, as well as reduced circulation CCK. Although saponins possess properties, such as large molecular weight, and hydrophilicity, leading to low bioavailability and poor penetration into central nervous system (35), there remains the possibility that the metabolism of saponins by intestinal flora yield metabolites possessing greater ability to cross the membrane barriers and exerts biological functions in the body (36, 37). However, the other biological functions of yucca in regulating feed intake cannot be excluded (e.g., antioxidant capacity especially in the hypothalamus) (2, 16).

The oxidative stress in broiler chickens was induced by HT, as reflected by the increased plasma lipid oxidation end product-MDA. However, GSH was increased by HT, which may reflect the biological feedback in response to heat stress. Interestingly, yucca supplementation reduced the lipid oxidative indicators, indicating its antioxidant capacity. Unexpectedly, the plasma antioxidative enzymes (Gpx and SOD) were not altered by yucca although it was reported previously that dietary supplementation with yucca schidigera extract alleviated the heat stress-induced oxidative damage through up-regulating antioxidant enzymes in Nile tilapia (38). Similar to our study, there were reports showing that no effects of saponin were observed on antioxidant enzymes (e.g., SOD and Gpx), while lipid hydroperoxide decreased in rats (39). Based on these results, it can be assumed, that yucca may exert antioxidants probably through a direct antioxidant activity rather than modulation of an antioxidant enzyme, because of the chemical structure of saponin (40).

## Conclusions

In summary, HT induced an obvious reduction in feed intake of broilers, probably involving in the modulation of circulation and hypothalamic peptides, while dietary supplementation of yucca could alleviate heat stress, and improve feed intake probably through down-regulating CCK in plasma and hypothalamus. Also, yucca supplementation attenuated the HT-induced increase of body temperature, probably through regulating hypothalamic temperature sensing genes. Our results indicate that supplementation of yucca serves an excellent option for future phytogenic feed additives in poultry especially in circumstances where stress is induced.

## Data Availability Statement

The original contributions presented in the study are included in the article/supplementary material, further inquiries can be directed to the corresponding author/s.

## Ethics Statement

The animal study was reviewed and approved by Animal Welfare Committee of Institutes of Animal Sciences, Guangdong Academy of Agricultural Sciences (IASCAAS2022). Written informed consent was obtained from the owners for the participation of their animals in this study.

## Author Contributions

JL and JW was responsible for designing and conducting the study. WC and JW was responsible for data analysis and interpretation, drafting of the manuscript, and approval of the submitted manuscript. HQ, YL, DS, and JW was responsible for the conception of the study and manuscript writing and revisions. CL and JJ was responsible for acquisition of data and manuscript revision. All authors contributed to the article and approved the submitted version.

## Funding

This work was supported by the National Key Research and Development Program, P. R. China (Grant No. 2018YFE0128200), the Science and Technology Program of Guangdong Province, P. R. China (Grant No. 2020B0202090004), Foreign Expert Project, P. R. China (QNL20200130001), Special fund for scientific innovation strategy-construction of high level Academy of Agriculture Science (R2020PY-JX006, R2020PY-JX008, and 202107TD), and the China Agriculture Research System of MOF and MARA (CARS-41).

## Conflict of Interest

YL was employed by the company Dekang Group Co. Ltd. The remaining authors declare that the research was conducted in the absence of any commercial or financial relationships that could be construed as a potential conflict of interest.

## Publisher's Note

All claims expressed in this article are solely those of the authors and do not necessarily represent those of their affiliated organizations, or those of the publisher, the editors and the reviewers. Any product that may be evaluated in this article, or claim that may be made by its manufacturer, is not guaranteed or endorsed by the publisher.
